# Unraveling the Mechanisms of Ch-SeNP Cytotoxicity against Cancer Cells: Insights from Targeted and Untargeted Metabolomics

**DOI:** 10.3390/nano13152204

**Published:** 2023-07-29

**Authors:** Hector Estevez, Estefania Garcia-Calvo, Maria L. Mena, Roberto Alvarez-Fernandez Garcia, Jose L. Luque-Garcia

**Affiliations:** Department of Analytical Chemistry, Faculty of Chemical Sciences, Complutense University of Madrid, 28040 Madrid, Spain; hestevez@ucm.es (H.E.); egcalvo@ucm.es (E.G.-C.); mariluz@ucm.es (M.L.M.); robalvar@ucm.es (R.A.-F.G.)

**Keywords:** selenium nanoparticles, cancer, metabolomics, cell-cycle arrest, TCA cycle, hypoxia

## Abstract

Although chitosan-stabilized selenium nanoparticles (Ch-SeNPs) have emerged as a promising chemical form of selenium for anticancer purposes, gathering more profound knowledge related to molecular dysfunctions contributes significantly to the promotion of their evolution as a chemotherapeutic drug. In this sense, metabolites are the end products in the flow of gene expression and, thus, the most sensitive to changes in the physiological state of a biological system. Therefore, metabolomics provides a functional readout of the biochemical activity and cell state. In the present study, we evaluated alterations in the metabolomes of HepG2 cells after the exposure to Ch-SeNPs to elucidate the biomolecular mechanisms involved in their therapeutic effect. A targeted metabolomic approach was conducted to evaluate the levels of four of the main energy-related metabolites (adenosine triphosphate (ATP); adenosine diphosphate (ADP); nicotinamide adenine dinucleotide (NAD^+^); and 1,4-dihydronicotinamide adenine dinucleotide (NADH)), revealing alterations as a result of exposure to Ch-SeNPs related to a shortage in the energy supply system in the cell. In addition, an untargeted metabolomic experiment was performed, which allowed for the study of alterations in the global metabolic profile as a consequence of Ch-SeNP exposure. The results indicate that the TCA cycle and glycolytic pathways were impaired, while alternative pathways such as glutaminolysis and cysteine metabolism were upregulated. Additionally, increased fructose levels suggested the induction of hypoxia-like conditions. These findings highlight the potential of Ch-SeNPs to disrupt cancer cell metabolism and provide insights into the mechanisms underlying their antitumor effects.

## 1. Introduction

Metabolites are not only energy bricks for the cell but also signaling molecules, immune modulators, endogenous toxins, and environmental sensors, among others. All together, these chemical entities constitute the metabolome, which has had a relatively recent entry into the “multi-omics” tools. Metabolites are entwined in the metabolism machinery via the activity of genes and proteins. Thus, metabolomics is widely accepted as one of the most sensitive approaches for the discovery of key biomarkers and mechanisms for the understanding of pathophysiological processes.

Metabolomics, through analytical chemistry, enables the high-throughput characterization of metabolites from cells, organs, tissues, or biofluids. As technology improves, metabolomics is gaining more interest for unraveling the role that small molecules play in a great variety of biological processes. Consequently, metabolomics has been reconsidered as not only a biomarker identification tool, but also as an instrument that allows the discovery of active biological-process operators [1].

There is a crucial awareness about the role of metabolites that has changed the perception of how these small molecules participate in the evolutions of pathologies. Metabolites, far from being just a downstream product of gene or protein activity, actually show a wide range of regulatory activities related to other “omics” levels. Studies investigating seminal discoveries of relationships between glucose, fatty acids, and other lipids with insulin secretion, the lac operon in bacteria, and nutrient-sensing via the mammalian target of rapamycin (mTOR) kinase confirm the impact of metabolites on biological systems [2,3,4].

Generally, targeted metabolomics is focused on the identification and quantification of a small and defined chemically characterized group of known metabolites, providing information on a specific metabolic process. The use of internal standards allows for a quantitative or semi-quantitative analysis of the selected metabolites, and the dominance of abundant molecules as well as analytical artifacts can be avoided via the isolation of the target metabolites during sample preparation [5]. Here, our focal point was energy metabolism, due to its significance in cancer development. Therefore, we selected four key metabolites to dig into potential dysfunctions in the energy production machinery.

As a complementary approach, untargeted metabolomics is a major tool for global metabolite discovery when there is no prior specific metabolic hypothesis, comparing groups of samples and underlining perturbations between them. Therefore, these studies generate large quantities of data, which are not only characterized by their volume but also by their complexity [6,7].

Parallel to the evolution of metabolomics, nanomaterials have gathered a great deal of attention for their novel properties in comparison to larger-scale materials. In addition to a large number of applications, the integration of nanomaterials into chemotherapy is one of the most encouraging advances in nanomedicine (drug delivery, imaging, killing cancer cells, etc.) [8,9]. In previous studies by our group, selenium nanoparticles (SeNPs) proved their chemotherapeutic abilities in comparison to other selenospecies [10]. When hepatocarcinoma cells (HepG2) are exposed to Ch-SeNPs, they experience a drastic decrease in their normal proliferation. Based on their promising therapeutic effect, there is an urgent need for a deeper understanding of the molecular mechanisms through which these nanoparticles are capable of inhibiting cancer development. Metabolic signaling plays a central role in malignant transformation, cell proliferation, and cancer stemness, among others. Likewise, intercellular metabolite signaling modulates the inflammatory response in the tumor microenvironment. Discerning how SeNPs are responsible for metabolic-alteration outcomes is crucial for building a chemotherapeutic strategy based on this novel nanomaterial.

In the present work, in-house-synthesized chitosan-stabilized selenium nanoparticles (Ch-SeNPs) were characterized, and their cytotoxic effect in hepatocarcinoma cancer cells (HepG2) was investigated. Furthermore, the combination of both targeted and untargeted metabolomic experiments was proposed for an in-depth evaluation of the changes in the metabolic profile of the cancer cells due to exposure to Ch-SeNPs. The complementary results obtained by both approaches were in good agreement and exhaustively discussed, providing a deeper insight into the biomolecular mechanisms of action of these nanoparticles.

## 2. Materials and Methods

### 2.1. Synthesis and Characterization of Ch-SeNPs

Synthesis reagents, such as chitosan, ascorbic acid, acetic acid, and sodium selenite, were purchased from Sigma-Aldrich (St. Louis, MI, USA). Chitosan-stabilized selenium nanoparticles (Ch-SeNPs) were synthesized following the procedure described by Bai et al. [11]. Summarily, chitosan polysaccharide solution (0.5% *w*/*v*) was prepared using 0.5 M acetic acid. An amount of 0.25 mL of sodium selenite (0.51 M) was slowly added to a mixture of 10 mL of this chitosan solution, 7.5 mL of ascorbic acid (0.23 M), and 5 mL of acetic acid (2.4 M). A change in the solution from colorless to intense red was observed as a proof of the formation of the Ch-SeNPs. Then, the resulting colloidal suspension was diluted to 50 mL with deionized water (Millipore Corporation, Burlington, MA, USA), resulting in a final concentration of 200 mg/L of SeNPs and 0.1% of chitosan. An amount of 10 mL of the suspension was finally dialyzed for 2 h against 2 L of water, using a 12 kDa molecular-weight cut-off (MWCO) membrane (Merck, Darmstadt, Germany).

Ch-SeNPs were analytically characterized via transmission electron microscopy (TEM) and energy-dispersive X-ray spectroscopy (EDX) using a JEOL JEM 1400 PLUS operating at 120 kV and equipped with a charge-coupled-device CCD camera (KeenView Camera) (JEOL Ltd., Tokyo, Japan).

### 2.2. Cell-Culture and Exposure Conditions

For the in vitro study, the HepG2 cell line (ATCC HB-8065 TM, Manassas, VA, USA) was selected. The cells were grown at 37 °C and 5% CO_2_ in a Dulbecco’s modified Eagle’s medium (DMEM) supplemented with 10% fetal bovine serum (FBS) and antibiotics (penicillin: 50 U/mL; streptomycin: 50 U/mL). Cells were exposed to different concentrations of Ch-SeNPs. The appropriate concentration of Ch-SeNPs was suspended in DMEM and added to the cell culture. Cells were then incubated for 72 h at 37 °C and 5% CO_2_.

### 2.3. Cell Viability

The 3-(4,5-dimethylthiazol-2-yl)-2,5-diphenyltetrazolium bromide (MTT) assay was chosen for the evaluation of the effects on the cellular viability after Ch-SeNP exposure. The MTT assay measures the metabolic activity of viable cells, which are able to reduce the MTT to formazan (a purple-colored compound). Cells were seeded in 96-well plates at a concentration of 9 × 10^3^ cells per well. After cell attachment, they were exposed to 0.1, 0.5, 1, and 5 mg/L of Ch-SeNPs for 72 h. Then, 20 µL of MTT solution (5 mg/mL) was added to each well and incubated for 4 h at 37 °C. The formed formazan crystals were dissolved in 100 μL of dimethyl sulfoxide, and the absorbance of the resulting solution was measured at 595 nm in a microplate reader (Sunrise, Tecan, Männedorf, Switzerland).

### 2.4. Targeted Metabolomic Analysis

#### 2.4.1. Extraction of Energy-Related Metabolites

HepG2 cells were cultured in 100 mm Petri dishes and exposed to Ch-SeNPs at a concentration of 1 mg/L for 72 h. Following the treatment, both the exposed and control cells underwent a washing step using 0.9% (*w*/*v*) sodium chloride (Fisher Scientific, Waltham, MA, USA) and were then placed on ice. To extract metabolites, 100 µL of methanol at −20 °C and 400 µL of water containing 2% (*v*/*v*) formic acid (Fisher Scientific, Waltham, MA, USA) were added to the cells before harvesting. The cells were transferred to Eppendorf^TM^ tubes and vortexed for 1 min. After 3 min of incubation on ice, 45 μL of 15% ammonium bicarbonate (*w*/*v)* was added to neutralize the pH of the samples. The samples were vortexed for 1 min, incubated for 20 min on ice, and then centrifuged at 16,000× *g* and 4 °C for 10 min. The supernatant was collected and filtered through a Whatman^®^ PTFE membrane filter (pore size: 0.22 μm; Merck, Darmstadt, Germany). The total protein concentration of each sample was determined using the Bradford assay, allowing for the normalization of the concentration of each metabolite to account for variations in the initial cell content. Ten replicates were used for each studied condition, including samples treated with Ch-SeNPs and untreated controls.

#### 2.4.2. Mass Spectrometry (LC-QqQ-MS) Analysis

The investigation was performed using an LC-QqQ-MS instrument (LC/MS-8030 Shimadzu, Kyoto, Japan) that was equipped with an electrospray ionization source (ESI) operating in the negative-ionization mode. The quantification of four metabolites related to energy: ATP, ADP, NADH, and NAD+, was carried out using the Multiple-Reaction-Monitoring (MRM) mode, following the method described by García-Calvo et al. [12]. The MRM chromatograms for each analyte in both the control and Ch-SeNP-exposed samples can be observed in Figure S1.

#### 2.4.3. Statistical Analysis

Variances in the ATP, ADP, NADH, and NAD+ levels between the control group and cells exposed to Ch-SeNPs were evaluated using ANOVA statistical tests with a confidence level of 95% (*p*-value < 0.05). Subsequently, a Bonferroni test was conducted for further analysis.

### 2.5. Untargeted Metabolomics

#### 2.5.1. Sample Preparation

HepG2 cells were cultured in 100 mm Petri dishes and exposed to Ch-SeNPs at a concentration of 1 mg/L for 72 h. Following the exposure, the cell-culture medium was removed, and the cells were washed with 10 mL of 0.9% NaCl. To halt cell metabolism, a mixture of 400 μL of methanol at −20 °C and 400 μL of ice-cold water was added to the cells. The cells were then scraped and transferred into 1.5 mL tubes.

For metabolite extraction, an ultrasound-assisted method was employed. This involved adding 400 μL of chloroform at −20 °C to the tubes, followed by subjecting them to 20 sonication pulses (2 s pulse and 5 s rest) using a cell disruptor (FB50, Fisher Scientific, Waltham, MA, USA). The samples were kept on ice during the extraction process. Subsequently, the tubes were centrifuged at 13,000 rpm and 4 °C for 5 min, resulting in the separation of two distinct phases (polar and non-polar). From each phase, 300 μL was transferred to separate glass vials.

For further analysis, the protein interphase was isolated and quantified using the Bradford method. In each of the phases, 20 mg/L of 4-phenylbutyric acid (Sigma-Aldrich, St. Louis, MI, USA) was added as an internal standard. The extracts were then evaporated under a nitrogen stream at 4 °C. To prepare the samples for gas chromatography–mass spectrometry (GC-MS) analysis, metabolites were chemically derivatized. The samples were reconstituted in 30 μL of 40 mg/L methoxyamine hydrochloride in pyridine (Sigma Aldrich, St. Louis, MI, USA) and mixed at 500 rpm for 90 min at 37 °C. Afterward, 60 μL of N,O-Bis(trimethylsilyl) trifluoroacetamide (BSTFA) (Sigma Aldrich, St. Louis, MI, USA) containing 1% trimethylsilyl chloride (TMCS) (Sigma Aldrich, St. Louis, MI, USA) was added and incubated at 60 °C with mixing at 500 rpm for 1 h [13].

To ensure the reliability and stability of the metabolomic approach, quality-control (QC) samples were prepared. The residual supernatants from all samples were pooled and divided into 200 μL aliquots. These QC samples underwent the same treatment as the analytical samples during vacuum-drying, resuspension, instrumental analysis, and data processing. For every five analytical samples, one QC sample was inserted and analyzed.

#### 2.5.2. High-Resolution Mass spectrometry (GC-TOF-MS) Analysis

Ten replicates of each experimental condition, including samples exposed to Ch-SeNPs and untreated controls, were subjected to analysis using a gas chromatograph (7890A Agilent, Santa Clara, CA, USA) coupled to a high-resolution time-of-flight mass spectrometer (GCT Premier Micromass Waters, Waters Corporation, Milford, MA, USA).

During the analysis, a 2 μL volume of the samples was injected in split mode at a 1:3 ratio. Metabolite separation was achieved using a ZB-5MS plus column (30 m, 0.25 mm × 0.25 μm; Phenomenex, Alcobendas, Spain) at a flow rate of 1 mL/min, with *He* serving as the carrier gas. The column temperature was initially set at 60 °C for 3 min, and then it was gradually increased at a rate of 6 °C/min until reaching 325 °C, where it was held for 3 min. The inlet temperature was maintained at 270 °C, and a solvent delay of 2.5 min was employed.

The mass spectrometer was equipped with an electron-ionization (EI) ion source, and it recorded spectra within a mass range of from 50 to 800 m/z. To monitor the performance of the analysis, a GC-column test standard (Phenomenex, Alcobendas, Spain) was included and analyzed every 5 samples as a quality-control measure.

#### 2.5.3. Data Treatment and Statistical Analysis

The chromatographic data obtained from the analysis were processed using Mass Lynx software. For each metabolite, the peak area was determined and then normalized based on the internal standard. Metabolites were identified by analyzing their mass spectra and accurate masses using the NIST MS search 2.0 library.

For multivariate statistical analysis, the data were autoscaled using the mean and standard deviation of the entire dataset [14]. Pearson’s correlation analysis was performed using Microsoft Excel software. Additionally, principal component analysis (PCA) was conducted using Unscrambler software (version 9.7) as an unsupervised technique to reduce the dataset’s dimensionality and identify patterns.

The data matrix was established with metabolites as columns and HepG2 cells exposed to Ch-SeNPs and control cells as rows. To compare the normalized peak areas of the metabolites between the two experimental conditions, a two-tailed Student’s *t*-test was employed at a 95% confidence level (*p*-value < 0.05 considered statistically significant).

Metabolites that showed statistically significant differences in levels after Ch-SeNP exposure (*p*-values < 0.05) were used to construct a network using STITCH software (version 4.0). The network was created with a medium stringency of 0.40 and allowed the inclusion of some proteins as predicted functional partners with a score of up to 0.99.

Finally, the network was visualized using Cytoscape software (version 3.7.0) to gain insights into the interactions and relationships among the identified metabolites and proteins, shedding light on the effects of Ch-SeNP exposure on the cellular metabolic pathways.

## 3. Results

### 3.1. Characterization of Ch-SeNPs

**Figure 1:**
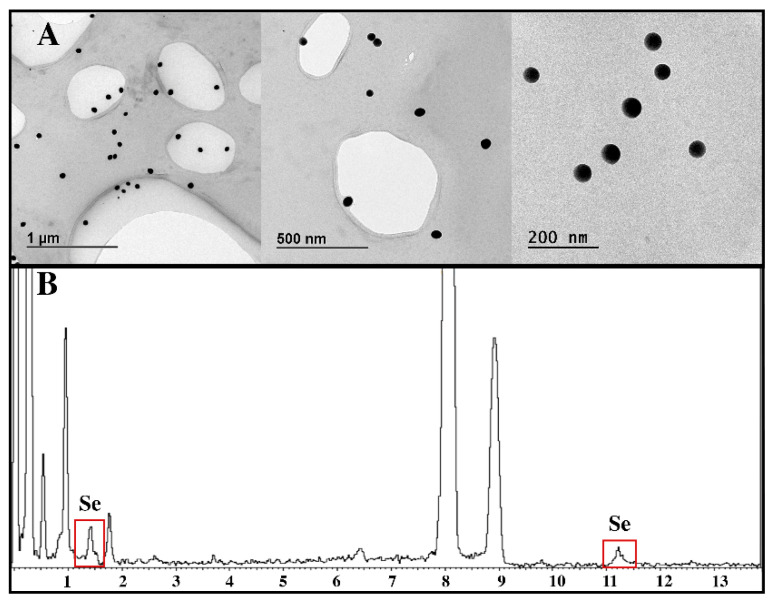
(**A**) TEM micrographs of well-dispersed Ch-SeNPs with sizes ranging from 40 to 50 nm; (**B**) EDS analysis.

As shown in Figure 1A, the followed Ch-SeNP-synthesis strategy provided spherical and well-dispersed nanoparticles, with sizes ranging between 40 and 50 nm. These features make them ideal for their internalization by HepG2 cells in culture. An EDS analysis (Figure 1B) assured the presence of selenium in the samples.

### 3.2. Cytotoxicity of Ch-SeNPs

**Figure 2:**
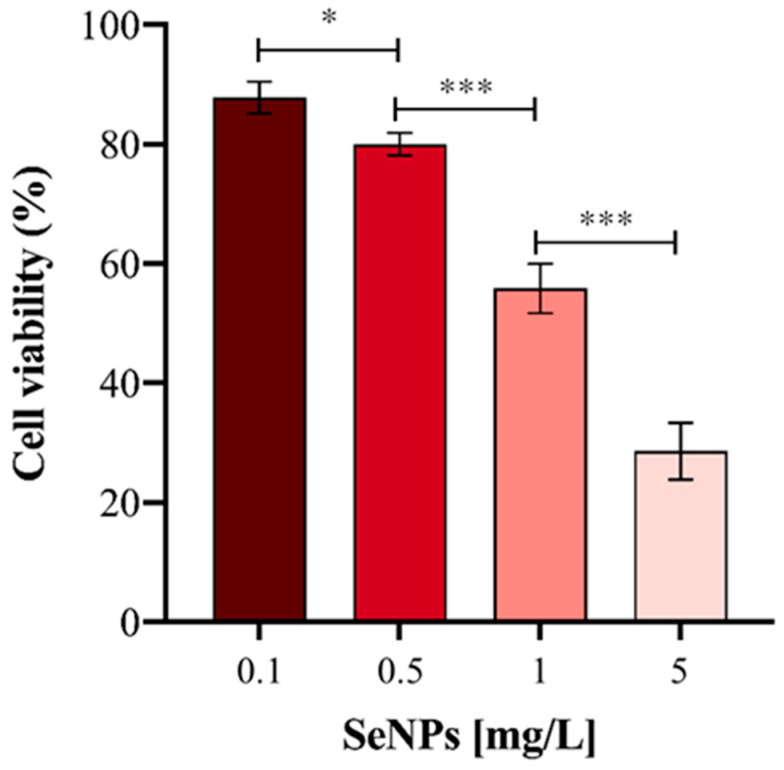
Cell viability (%) of HepG2 cells exposed to different concentrations of Ch-SeNPs. Results are plotted as the mean ± relative standard deviation (*n* = 5, statistical significance: * *p* < 0.05, *** *p* < 0.001).

Before carrying out the metabolomic approach, and in order to confirm previous results, the viability of the HepG2 cells was evaluated after exposure to Ch-SeNPs for 72 h. As expected, the cell viability decreased in a concentration-dependent manner (Figure 2). A decrease in cell viability close to 70% was obtained for the highest concentration of particles assayed (5 mg/L). However, for the targeted and untargeted metabolomic experiments, a concentration of 1 mg/L was chosen in order to further investigate the antitumor effect of the particles without drastically compromising the cell viability.

### 3.3. Evaluation of Levels of Energy-Related Metabolites

Analysis via LC-QqQ-MS working in MRM mode allowed for the quantification of the selected metabolites (ATP, ADP, NADH, NAD^+^) in the control and treated samples in order to unravel energy metabolism alterations as a consequence of Ch-SeNP exposure.

**Figure 3:**
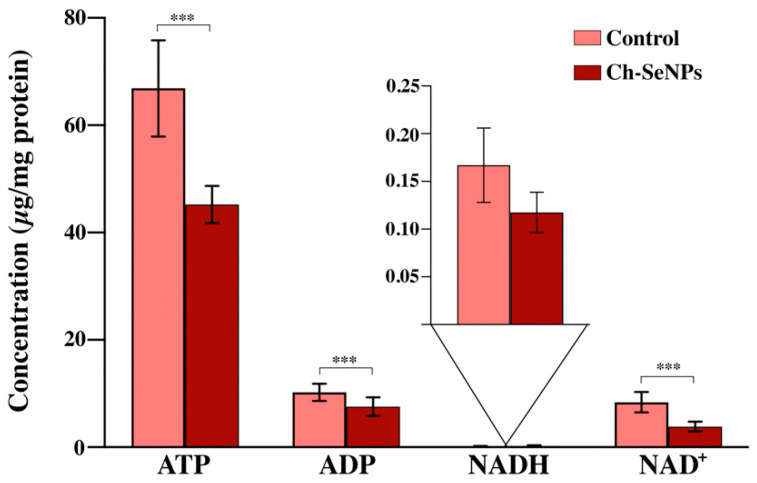
Concentration levels for ATP, ADP, NADH, and NAD^+^ in HepG2 cells exposed to Ch-SeNPs (1 mg/L). Results are plotted as the mean ± relative standard deviation (*n* = 10). The data were evaluated via ANOVA and subsequent Bonferroni test (*** *p* < 0.001; ns: non-significant).

As Figure 3 illustrates, significant differences (with a 95% confidence level, *p*-value < 0.05) in the concentrations of ATP, ADP, and NAD^+^ were found. The levels of these metabolites were found to be significantly diminished in the Ch-SeNP-exposed cells in comparison with the control cells. On the contrary, no significant differences were found in the analysis of the NADH levels of the non-exposed cells in comparison to the Ch-SeNP-exposed cultures.

### 3.4. Identification of Metabolic Alterations in Cells Exposed to Ch-SeNPs

**Figure 4:**
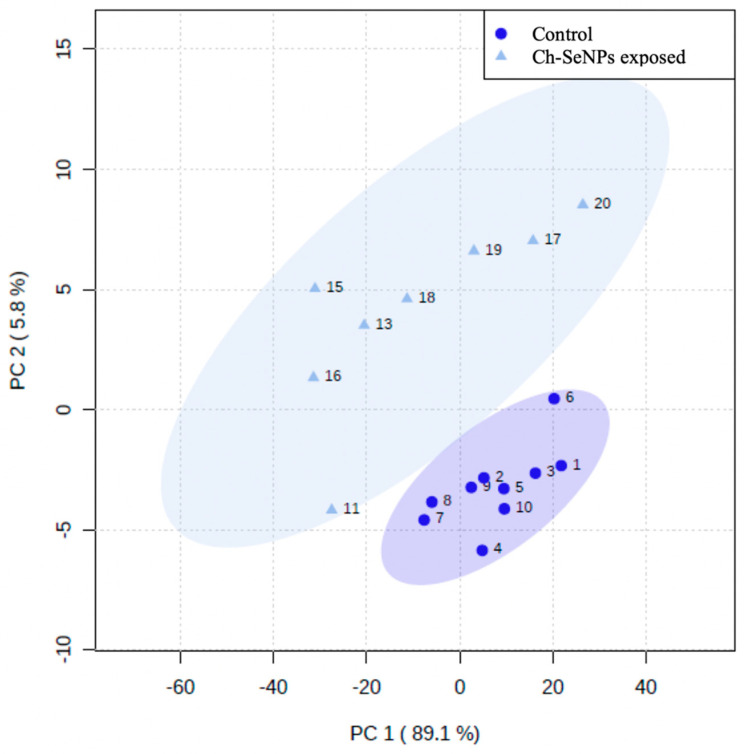
Principal component analysis for control and Ch-SeNP-exposed cells.

The GC-TOF-MS analysis allowed for the identification (minimum NIST Rmatch of 700) of 36 common metabolites, from a wide range of biochemical natures, between the treated and control cells. A multivariant analysis, by means of principal component analysis (PCA), was required to analyze the data [15]. The projection of the obtained data for each sample in the first two principal components allowed for the visualization of the groups and the interrelations between them. As shown in Figure 4, PC1 and PC2 represented 94.9% of the explained variance. A clear separation could be appreciated between the control and Ch-SeNP-treated samples, which reflects the different metabotypes between both experimental conditions.

Posterior statistical analysis based on the Student’s *t*-test detected significant differences (*p*-values < 0.05) in the average normalized areas and, thus, in the concentration levels of the metabolites from the exposed and control cells. Among the 36 identified metabolites, 13 were shown to be upregulated in the Ch-SeNP-exposed cells, while 13 metabolites had significantly lower concentrations. Table 1 gathers the retention time and “NIST Rmatch” parameter for each metabolite, in addition to the ratio between the average normalized areas.

**Figure 5:**
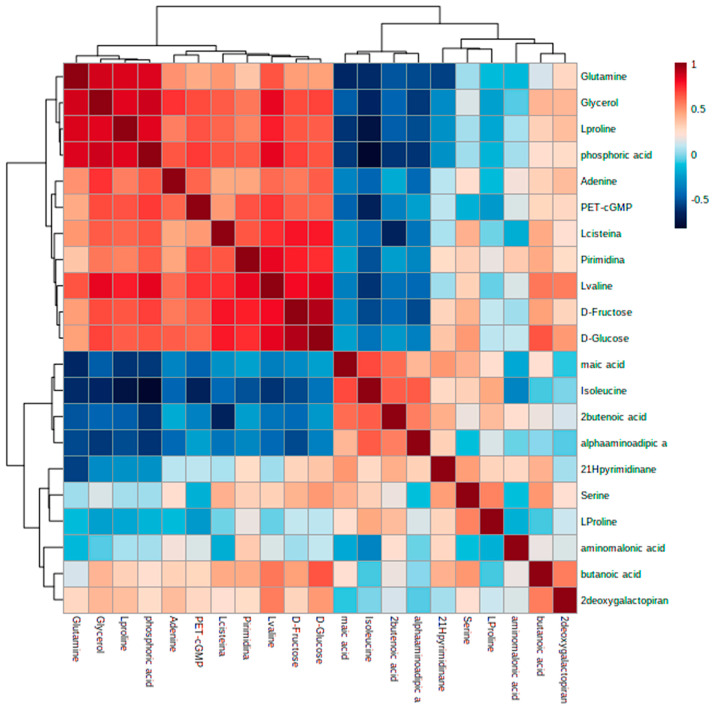
Hierarchical cluster analysis and heatmap of metabolites found at different levels (represented by colors) in control vs. treated samples (*p* < 0.05).

As a tool for interrelation comparison, a hierarchical-clustering and heatmap graphical representation is represented in Figure 5 in accordance with the results described.

## 4. Discussion

Commonly synthesized bare selenium nanoparticles are prone to aggregate in aqueous solutions, hindering their uptake by cells and reducing their bioavailability. As previously shown by Luo et al. and Zhang et al. [16,17], the encapsulation of selenium nanoparticles into a polysaccharide-like chitosan significantly improves their antioxidant properties and their retention in the cells. Here, chitosan enwraps selenium nanoparticles by means of hydrogen bonds between the hydroxyl groups of chitosan and selenite. The use of chitosan under the synthesis conditions allowed for control over the size and morphology of the particles, obtaining well-dispersed and spherical Ch-SeNPs with sizes ranging from 40 to 50 nm, as seen in Figure 1. A dialysis was performed to remove all the reagent excess from the final Ch-SeNP suspension, as a guarantee that the observed effects in the metabolism alterations were only related to Ch-SeNP exposure.

The synthesized Ch-SeNPs were able to significantly reduce the viability of the HepG2 cells after 72 h, showing their antitumoral potential (Figure 2). Based on these results, 1 mg/mL of Ch-SeNPs was selected as the optimum concentration for carrying out the metabolomic approaches, as the cells were affected but not drastically compromised.

To evaluate alterations in energy metabolism after Ch-SeNP exposure, four energy-related metabolites were analyzed: ATP, ADP, NADH, and NAD^+^. Energy within cells is obtained from the oxidation of carbohydrates, lipids, or proteins, among others. Free energy derived from oxidation processes is stored in phosphoanhydrine “high energy bonds” within molecules such as adenosine 5’diphosphate and adenosine 5’triphosphate (ADP and ATP, respectively). The four measured metabolites participate in the three main steps of cellular respiration: glycolysis, the TCA cycle, and the mitochondrial electron transport chain.

LC-QqQ-MS working in MRM mode presents high sensitivity and selectivity; thus, it was the analytical platform selected for quantifying these metabolites. Analytical features of the LC-QqQ-MS method employed were previously optimized by García-Calvo et al. [12]. The four metabolites were quantified both in the control and in cells exposed to Ch-SeNPs. Each concentration was normalized based on the total protein concentration of each sample, previously determined via a Bradford assay. Statistical ANOVA assays were conducted with a 95% confidence level (*p*-value < 0.05), and Bonferroni’s post-test was performed. Significant differences in the concentrations of ATP, ADP, and NAD^+^ between the control and Ch-SeNP-exposed cells were observed (Figure 3). The concentrations of these three energy-related metabolites were significantly lower as a result of the Ch-SeNP effect. However, no significant differences were observed for the NADH levels.

Anabolic pathways will consume ATP and generate NADH, while catabolic pathways will generate ATP from ADP + Pi and oxidize NADH into NAD^+^. Therefore, ATP and NAD^+^ are key molecules in growth processes, depending on the ATP synthesis and NADH turnover [18]. Cancer cells, in order to sustain abnormal proliferation, require a more significant amount of energy production than healthy cells. Mostly, metabolic reprogramming in cancer is characterized by enhanced glucose uptake and glycolysis, generating larger amounts of ATP in the cytosol. In sufficient oxygen-concentration conditions, the rate of oxidative phosphorylation is governed by the mitochondrial ATP synthase, which catalyzes the synthesis of ATP through the proton-motive force generated by the respiratory chain [19,20]. ATP, as the central energy-storing molecule in the cell, is responsible for fueling the majority of biological processes. In our experiment, one of the most significant results of the HepG2 cells after Ch-SeNP exposure was the drastic reduction in the ATP levels. As commented above, energy supplies depend directly on this molecule; therefore, in cancer cells, it is even more critical to prevent uncontrolled proliferation.

In addition, in order to complete aerobic glycolysis, NAD^+^ has to be regenerated from NADH in the pyruvate–lactate step. NADH, produced by glyceraldehyde phosphate dehydrogenase (GAPDH), is consumed, regenerating NAD^+^ and keeping glycolysis active. Higher glycolysis conversions are yielded in biomolecule-production-line activity, such as the conversion of siphon 3-phosphoglycerate to serine for the one-carbon metabolism-mediated production of nucleotides [21,22]. After Ch-SeNP exposure, the NAD^+^ levels were reduced by approximately 50%, which was directly related to the glycolytic activity and, thus, to a metabolic situation that prevented the cancer cells from meeting their biosynthetic requirements for uncontrolled proliferation.

To obtain a deeper insight into the metabolic alterations experienced by cells exposed to Ch-SeNPs, an untargeted metabolomic approach based on GC-MS was carried out. A total of 36 metabolites were identified, including fatty acids, amino acids, organic acids, sugars, and other small molecules. As shown in Figure 4, while samples within the same experimental condition presented similar metabolic profiles and were grouped together, a clear separation between the control and Ch-SeNP-exposed cells was obtained, reflecting metabolic differences between the two conditions (Figure 5).

The statistical analysis revealed significant alterations in 26 metabolites (Table 1), of which 13 were at significantly higher concentrations in the exposed cells, while the other 13 were at lower levels compared to the control cells.

The promising potential of Ch-SeNPs in cancer therapy through cell-cycle arrest has been observed in previous studies [8]. The inhibition of tyrosine (R_M_ = 0.18), in our study, supports these results, as it is involved in the activation of protein tyrosine phosphatase-signaling events. The downregulation of this protein activity decreases cell proliferation, as well as the expressions of cyclins E and B1, PCNA, PTTG1, and phospho-histone H3 proteins, and, consequently, causes cell-cycle arrest [23]. High levels of palmitic acid, also known as hexadecanoic acid, (R_M_ = 2.87), have been reported to induce cell-cycle arrest and promote apoptosis in human neuroblastoma, prostate cancer, and breast cancer cells, which is in accordance with the effects observed in cells exposed to Ch-SeNPs [24].

Cancer metabolism is constantly reprogrammed to enable abnormal proliferation and to ensure survival under insufficient-nutrient environments. Given that proline (R_M_ = 0.47), serine (R_M_ = 0.18), and glycine (R_M_ = 0.25) are essential for tumor growth and survival, they are considered oncogenesis-supportive metabolites; all of them were found to be downregulated in cells exposed to Ch-SeNPs [25,26]. Some studies have directly linked proline biosynthesis to the tumor-growth capacity [27]. The other two amino acids are closely related, as serine interconversion provides glycine, generating a large number of one-carbon units. These units induce one-carbon metabolism, which is key in cancer metabolism for the methylation pathways and biosynthesis of both purine and pyrimide nucleotides, among others [28]. These one-carbon units fuel two different pathways: the folate cycle [29,30] and the methionine cycle [31]. Therefore, Ch-SeNPs appear to be responsible for draining serine and glycine and prevent HepG2 cells from using them as a molecular base for the synthesis of organic molecules or as energetic agents [32,33,34,35]. Consequently, alterations in serine and glycine metabolism result in profound dysregulations in normal cancer progression. Following on from this idea, the downregulation of alpha-aminoadipic acid (R_M_ = 0.01) is also related to tumor shrinkage through the activation of a tumor suppressor known as KLF4 [36]. In addition to all this, in cancer cells, lipids are essential for maintaining the integrity of biological membranes and providing energy for malignant biological behavior. The accumulation of glycerol (R_M_ = 3.18), as a decrease in its transformation to glycerol-3-phosphate, the main intermediate for generating lipids and ATP, also hinders the proliferation of cancer cells [37].

The bioenergetic profiles of tumors can provide crucial prognostic information, raising the need for targeted therapies against the primary pathways of energy production. The energy imbalance produced by Ch-SeNPs was reflected in the decreased levels of ATP, ADP, and NAD^+^, as shown in the targeted metabolomic results. In fact, ATP, together with adenosine (R_M_ = 0.11), fuel signaling pathways that are thought to promote cancer growth and malignancy. The inhibition of both metabolites is in agreement with the paralysis of cancer cell growth [38]. One of the metabolic pathways that provides energy and intermediates for biosynthesis is the tricarboxylic acid (TCA) cycle. Glucose is, in a first step, oxidized into pyruvate in the cytoplasm, and the complete metabolism of glucose takes place through the TCA cycle. The ATP production in the TCA cycle occurs via NADH and FADH2 as intermediates after oxidative phosphorylation (OXPHOS) [21,39]. The downregulation of both ATP and NAD^+^ seems to reflect the inhibition of both pathways. The alteration of different metabolites, following the study of the untargeted metabolomic results, confirmed this hypothesis. For example, 2-hydroxybutanedioic acid or malic acid, which is an intermediate of the TCA cycle necessary for energy production through the consumption of pyruvic acid [40,41,42,43], was found at lower levels in the cells exposed to Ch-SeNPs (R_M_ = 0.15). Another metabolite that revealed the impairment of TCA-cycle progression was pantothenic acid (R_M_ = 0.11), an essential vitamin (B5) that plays a central role in the synthesis of CoA and, therefore, in the TCA cycle and fatty acid metabolism. Also, CoA serves as a precursor for acylation reactions. CoA catabolism generates pantotheine, which is acted upon by vanins (pantotheinases), which generate pantothenate and cysteamine [44]. After Ch-SeNP exposure, the HepG2 cells showed drastically lower levels of pantothenic acid in comparison with the non-treated cells, which leads to the conclusion that the exposure to Ch-SeNPs impairs the TCA cycle and, therefore, reduces the production of energy in the cell, which is essential for the survival of cancer cells.

**Figure 6:**
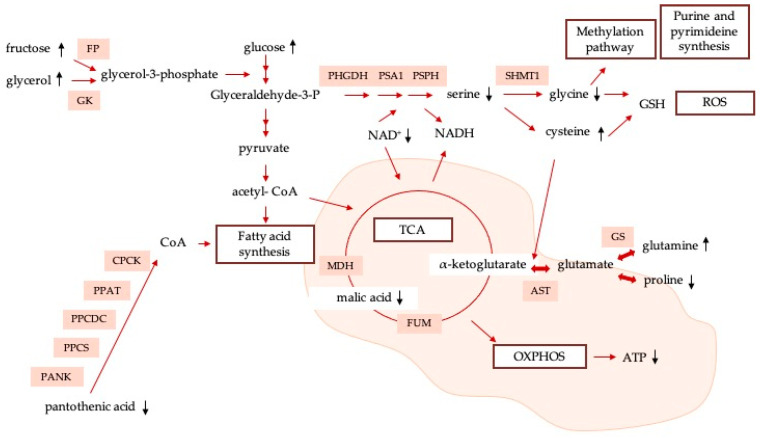
Network map containing main metabolic pathways involved in antitumoral effect of Ch-SeNPs.

Although being a relevant route in terms of oxidative phosphorylation, it has been observed that, under certain circumstances, cancer cells bypass the TCA cycle and predominantly use aerobic glycolysis for fulfilling their bioenergetic, biosynthetic, and redox-balance requirements. Recent evidence suggests that tumor cells are able to uncouple glycolysis from the TCA cycle, allowing the use of glutamine (R_M_ = 4.85) to meet their needs [45]. Specifically, cancer cells are normally characterized by significantly promoting glutaminolysis in comparison to healthy cells, giving glutamine a central role in cancer proliferation [46,47]. Kamphorst et al. [48] demonstrated that KRAS-driven pancreatic cells scavenge proteins from the extracellular space for their lysosomal degradation in amino acids, such as glutamine, used to fuel the TCA cycle. In a similar way to what happens with glutamine, after Ch-SeNP exposure, HepG2 cells produce higher quantities of cysteine (R_M_ = 3.69) as compared to untreated cells. Cysteine is a proteinogenic amino acid with a free thiol group that confers particular properties on the functional sites of proteins. This amino acid plays key roles in the metabolic rewiring of cancer cells to withstand poor-nutrient and oxygen-deficient conditions. These roles are, for example, being a precursor of glutathione, regulating oxidative stress, and serving as a carbon source for biomass and energy production. In particular, cysteine is metabolized and converted into 3-mercaptopyruvate (3-MP), releasing one amino group that reacts with α-ketoglutarate, generating glutamate and pyruvate, and fueling, in an alternative way, the TCA cycle [49]. Higher levels of cysteine show that cells are rewiring their metabolisms in order to provide alternative ways of maintaining the normal functioning of the pathway, thus confirming that the main sources are altered due to Ch-SeNP exposure. Finally, fructose (R_M_ = 2.16) has been revealed as a strong modulator of metabolic syndrome, preferentially promoting the glycolytic pathway, and capable of inducing mitochondrial dysfunction and oxidative stress (suppressing aconitase in the TCA cycle) [50]. In addition to glucose, it has been suggested that fructose could also serve as an alternative nutrient for the growth of cancer cells. A scheme of the alteration of energy metabolism, previously described, is shown in Figure 6. It shows the main metabolites and metabolic pathways affected as a consequence of exposure to Ch-SeNPs, as well as the main proteins involved in them.

Increased fructose (R_M_ = 2.16) levels are also associated with other effects in the cell. Specifically, fructose metabolism is enhanced in response to low-oxygen or anoxia conditions, and it promotes the production of uric acid and lactate as the main metabolic products. Particularly, uric acid induces the Warburg effect by downregulating mitochondrial respiration and inducing aerobic glycolysis [51]. As observed by Park et al. [52], naked mole rats under hypoxia/anoxia endogenously produced substantial quantities of fructose in several organs, like the kidney and liver. In our experiment, fructose production appeared to be at significantly higher levels in the cells treated with Ch-SeNPs as compared to the control cells, which is a predictable fact in terms of cancer metabolism and energy sources. In addition, these higher levels of fructose observed in the treated cells correlate well with the fact that Ch-SeNP exposure has been previously demonstrated to induce hypoxia in cancer cells [53]. Furthermore, these results are in agreement with the targeted metabolomic results because fructose induces ATP depletion in a transient phenomenon, thereby reducing the intracellular ATP levels and impairing cellular growth. Another indicator of the hypoxia state in cells exposed to Ch-SeNPs might be the increased levels of cysteine (R_M_ = 3.69). Cysteine is a scavenger of free radicals, and it can abrogate most of the oxidative or alkylating drugs used in cancer therapy [49]; thus, the high levels of cysteine found as a cellular defense mechanism to help cancer cells survive drug exposure and other Ch-SeNP-mediated stress conditions, such as hypoxia, make perfect sense.

## 5. Conclusions

In this study, the combination of targeted and untargeted metabolomics provided valuable insights into the biomolecular mechanisms underlying the cytotoxic effects of Ch-SeNPs against cancer cells. The results confirm that Ch-SeNPs can disrupt cell-cycle modulators, reinforcing their potential for arresting cell growth and inhibiting cancer development. Several key metabolites crucial for cancer growth were found at lower levels in the treated cells compared to the controls, indicating the ability of Ch-SeNPs to restrict oncogenesis-supportive metabolites, such as proline, serine, and glycine.

Moreover, the targeted metabolomic experiment revealed significant alterations in energy metabolism following Ch-SeNP exposure. These nanoparticles were found to disrupt the TCA cycle, a primary source of cellular energy, leading to decreased levels of important energy-related metabolites, like pantothenic acid and malic acid. The cells adapted by shifting to glycolysis as an alternative pathway, as evidenced by the higher cysteine levels in the Ch-SeNP-exposed cells. Additionally, the study demonstrated the ability of Ch-SeNPs to induce hypoxia in cancer cells, as evidenced by the promotion of specific metabolites like fructose and cysteine, associated with this stress condition.

Compared to previous findings, this study offers new insights into the specific metabolic pathways targeted by Ch-SeNPs in cancer cells. The results reinforce the potential of Ch-SeNPs as an effective antitumor agent and shed light on the mechanisms underlying their therapeutic efficacy.

In conclusion, this research has advanced our understanding of the therapeutic potential of Ch-SeNPs and their mode of action against cancer cells. The findings suggest that Ch-SeNPs have a multifaceted impact on cancer metabolism, disrupting key pathways and restricting essential metabolites, which collectively contribute to their antitumor effects. These insights have implications for the development of targeted nanoparticle-based therapies in cancer treatment.

## Figures and Tables

**Table 1:** Significantly altered metabolites in HepG2 cells after exposure to 1 mg/L of Ch-SeNPs for 72 h.

Metabolite	Retention Time (min)	NIST Rmatch	R_M_ *
Glycine	9.56	844	0.25
Butanoic acid	9.64	783	1.84
Benzoic acid	10.14	718	1.50
L-Valine	11.55	848	0.18
Serine	12.47	798	0.61
Urea	12.63	825	1.35
Glycerol	12.91	896	3.18
L-Proline	13.33	729	0.47
Pyrimidine	14.17	816	1.50
2-butenoic acid	14.55	869	0.21
Aminomalonic acid	17.00	850	1.81
Malic acid	17.48	828	0.15
L-Cisteine	18.71	905	3.69
Glutamine	20.06	844	0.25
L-Isoleucine	20.74	747	0.16
Alpha-aminoadipic acid	21.79	732	0.01
Hydracrylic acid	23.39	863	1.97
2-desoxigalactopiranose	23.75	777	0.74
Adenine	24.25	824	1.20
D-Fructose	24.47	881	2.16
D-Glucose	24.89	817	1.64
Pantothenic acid	26.38	908	0.11
Inositol	26.95	854	0.22
N-acetylglucosamine	27.63	714	1.24
PET-cGMP	33.60	963	3.22
2-(1 H)-pyrimidinone	38.15	805	0.66

* Ratio exposed/control cells.

## Data Availability

Data is contained within the article or supplementary material.

## Supplementary Materials

The following supporting information can be downloaded at https://www.mdpi.com/article/10.3390/nano13152204/s1, Figure S1: MRM chromatograms of each analyte from both control and Ch-SeNP-exposed samples.
