# Supplementary figures and images for: Unraveling the Mechanisms of Ch-SeNP Cytotoxicity against Cancer Cells: Insights from Targeted and Untargeted Metabolomics
Source: Nanomaterials (Basel). 2023 Jul 29;13(15):2204. doi: 10.3390/nano13152204 (PMC10420838; doi:10.3390/nano13152204)

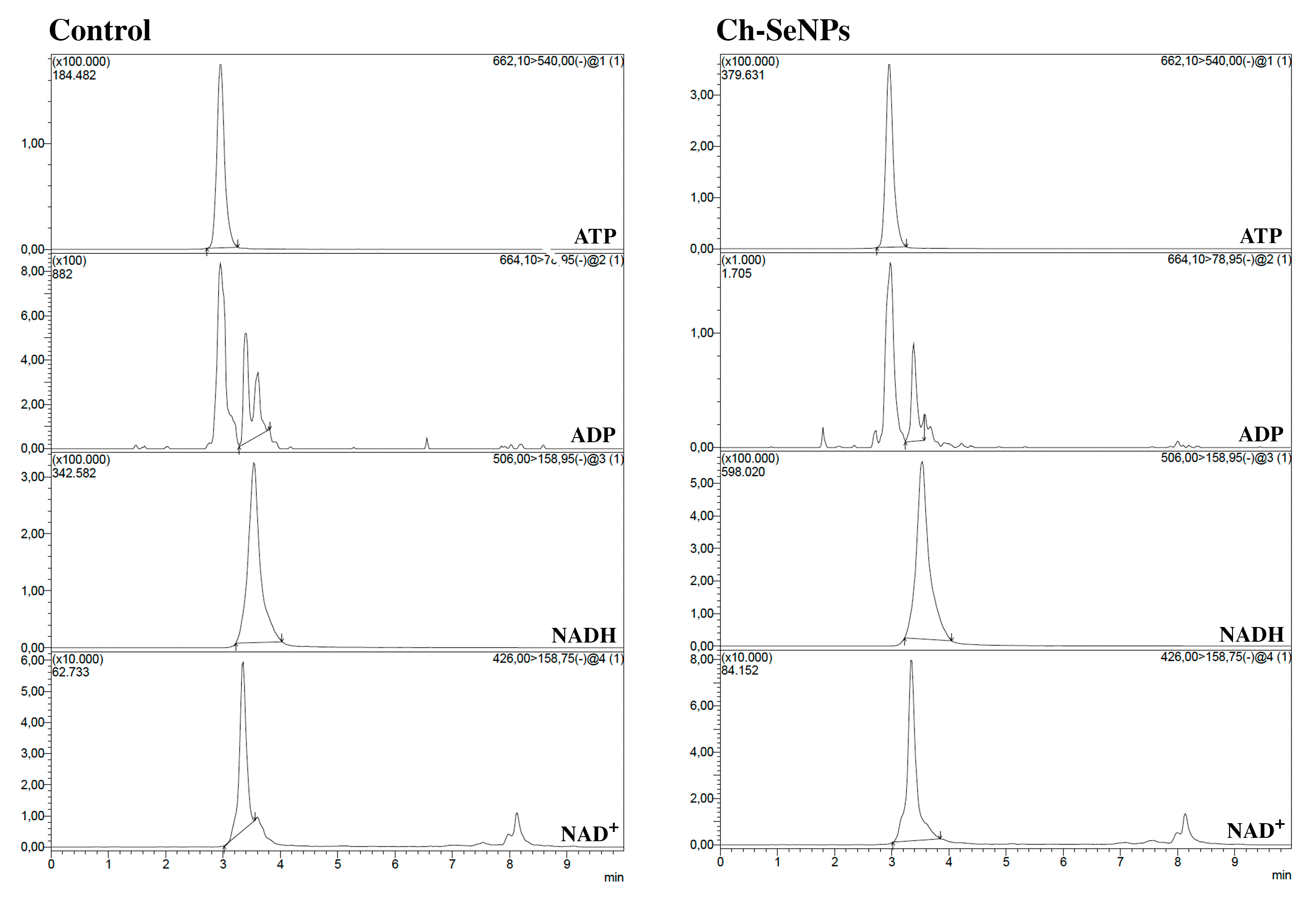

Supplement: Supplementary file 1 [file nanomaterials-13-02204-s001.zip › nanomaterials-2527418-supplementary.tif]
